# One Joint Aspirate: Three Diagnoses

**DOI:** 10.7759/cureus.17714

**Published:** 2021-09-04

**Authors:** Abdul Moiz Qureshi, Sara Tariq, Nismat Javed, Abu Baker Sheikh

**Affiliations:** 1 Internal Medicine, Shifa Tameer-E-Millat University Shifa College of Medicine, Islamabad, PAK; 2 Internal Medicine, Ascension Saint Agnes Hospital, Baltimore, USA; 3 Internal Medicine, University of New Mexico School of Medicine, Albuquerque, USA

**Keywords:** gout, pseudogout, reactive arthritis, chlamydophila pneumoniae, polyarthritis

## Abstract

Gout is a frequently diagnosed condition. However, it is rarely diagnosed with concomitant pseudogout or reactive arthritis (ReA) from ​​​​​*Chlamydophila pneumoniae (C. pneumoniae)*. This case report describes an interesting case of a 67-year-old man who presented with a two-week history of malaise, chills, and shortness of breath. He also reported a one-day history of polyarthritis, which limited his ambulation. The results of polarized microscopy revealed uric acid and calcium pyrophosphate crystals. The respiratory panel was positive for *C. pneumoniae* and rhinovirus. Therefore, he was diagnosed with gout, pseudogout, and ReA. Appropriate management led to a full clinical recovery. This is the first report documenting the simultaneous occurrence of ReA, gout, and pseudogout in a single patient. The association between these rheumatic diseases and a summary of similar cases in the literature are also discussed.

## Introduction

Reactive arthritis (ReA) can be defined as the development of sterile inflammatory arthritis as a sequel to remote infection. The diagnosis is mainly clinical and based on acute oligoarticular arthritis of larger joints developing within two to four weeks of the preceding infection [1]. *Chlamydophila pneumoniae (C. pneumoniae)* is an increasingly important pathogen, which is reported to be responsible for 10% of the pneumonia cases in the United States. Only 10% of diagnosed *C. pneumoniae* infections lead to a diagnosis of ReA. While the coexistence of gout and pseudogout has rarely been reported, Stockman et al. have investigated patients with gout and found that there were concomitant radiographic features of pseudogout in eight out of 138 (5.8%) patients in their series [2,3]. To our knowledge, the simultaneous occurrence of ReA, gout, and pseudogout has not been reported previously. We report the unusual case of a patient who was diagnosed with ReA as well as concurrent gout and pseudogout.

## Case presentation

A 67-year-old male presented to the emergency department with two weeks of malaise, chills, and shortness of breath. He also complained of a one-day history of asymmetric polyarthritis, which made it difficult for him to walk. He reported that the pain was worse in the left wrist and left knee. He had experienced a previous episode of similar pain that had resolved with long-term treatment with indomethacin and prednisone. He also had associated left knee swelling. He had been having complaints of flu-like syndrome for the last two weeks. He denied having any difficulties such as nausea, vomiting, constipation, diarrhea, rash, temporal tenderness, redness of eyes, dysuria, genital discharge, blurry visions, and neck stiffness. He also denied any falls. He also had a prior history of smoking.

On admission, he had a new oxygen requirement of 2L. The temperature was 36.3 °C, blood pressure was 125/68 mmHg, pulse was 89 bpm, respiratory rate was 20/minute, and oxygen saturation was 90% on 2L. On physical exam, he was found to have bilateral tenderness in his knees, wrist, elbows, hips, and shoulders. His lab workup revealed a white blood cell count of 9.3 x 10E^3^/μL, hemoglobin of 15 g/dL, hematocrit of 46%, and platelet count of 235 x 10E^3^/μL.

**Figure 1:**
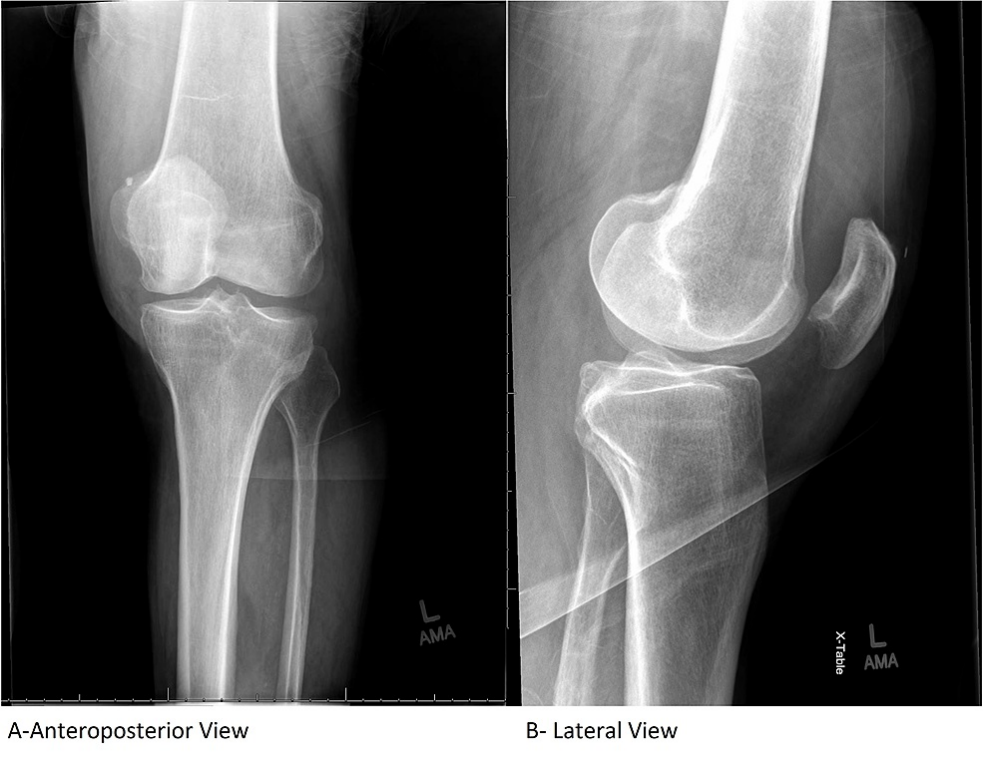
Left knee X-ray

The X-ray of the left wrist showed no osseous abnormality, but the X-ray of the left knee showed a large joint effusion with a radiopaque foreign body in the suprapatellar region (Figure 1).

**Figure 2:**
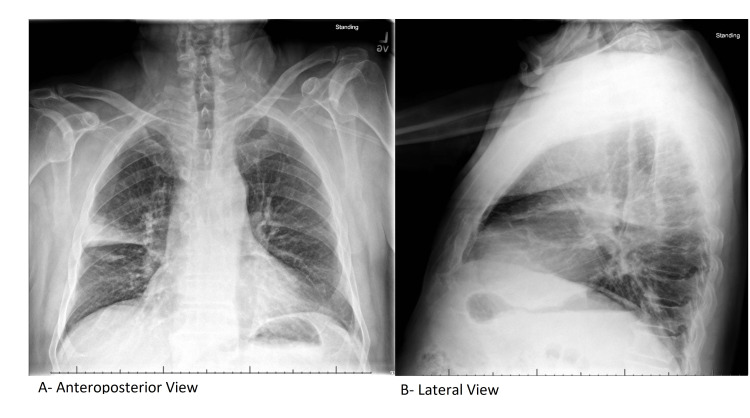
Chest X-ray

Orthopedic surgery was consulted to aspirate his left knee. Aspirate revealed uric acid crystals, calcium pyrophosphate crystals, 53,000 nucleated cells with 87% neutrophils, and an unremarkable gram stain. Additionally, a chest X-ray was ordered to evaluate the shortness of breath, which revealed right upper lobe pneumonia (Figure 2).

This was followed by a respiratory panel, which was positive for *C. pneumoniae* and rhinovirus. He was diagnosed with inflammatory arthritis secondary to gout and pseudogout as well as ReA secondary to *C. pneumoniae*.

He was started on an indomethacin course for one week with significant improvement in his arthralgias. For his *C. pneumoniae*, ceftriaxone and doxycycline were initially commenced followed by a de-escalation to doxycycline. He was discharged on azithromycin for a total five-day treatment course.

## Discussion

There are several interesting aspects to this case. This case represents the first reported occurrence of simultaneous gout, pseudogout, and ReA in the same joint. A search of MEDLINE/PubMed failed to show any previously reported cases of gout, pseudogout, and ReA in the same joint (Table 1).

**Table 1:** Previously reported cases of dual crystal arthropathy M: male; F: female; B/L: bilateral; CPP: calcium pyrophosphate crystals; MSU: monosodium urate crystals

Reference	Patient age/sex	Joints	Note
Wang et al. [2]	42/M	Right knee	Microscopic analysis of fluid revealed both MSU and CPP
Stockman et al. [3]		-	Eight cases were reported as part of a case-control study with concomitant gout and pseudogout
Gross et al. [4]	34/M; 59/F	Left temporomandibular joint; left temporomandibular joint	The tissue was unavailable for microscopic examination. Microscopic examination revealed CPP and MSU-shaped crystals
De Bari et al. [5]	63/M	Metatarsophalangeal joint of the left foot	Concomitant psoriatic arthritis, gout, and pseudogout
Colaco et al. [6]	67/M	B/L knee, shoulders, and right wrist	Joint aspirate showed uric acid and CPP crystals and group G streptococcus was isolated
Yoo et al. [7]	63/M	Right knee	Polarized microscopy showed both MSU and CPP crystals

ReA is mostly a clinical diagnosis based on oligoarthritis of larger joints that can develop within two to four weeks as a consequence of inflammation due to remote infection [1]. It is most commonly reported following gastrointestinal and genitourinary infections. Causative bacteria include Salmonellae, Shigella, *Campylobacter*, *Yersinia*, and *Chlamydia trachomatis*. There is also mounting evidence that *C. pneumoniae* is another etiology of ReA. *C. pneumoniae* is a common cause of atypical pneumonia or bronchitis and as many as 70% of infections are asymptomatic. In addition, when an acute infection with *C. pneumoniae* is symptomatic, a definitive diagnosis of this organism is often never established [8]. Only 10% of diagnosed *C. pneumoniae* infections lead to a diagnosis of ReA. While several laboratories have confirmed *C. pneumoniae* DNA and antigens in the synovial fluid of people with arthritis, it has been difficult to establish a cause-and-effect relationship. It is currently considered a “probable cause” of ReA [8]. The classic extra-articular manifestations associated with ReA from *C. trachomatis* are absent with *C. pneumoniae*. There have been several reports describing the coexistence of gout and pseudogout [2-6], but these are mostly single case reports. However, one case-control study did detect pseudogout in 5.8% of patients who suffered from gout (eight of 138) [3]. Overall, most diagnoses were made based on radiological surveys [3,5].

Classically, in pseudogout, crystals deposit in fibrocartilage, resulting in characteristic radiological linear calcifications [9]. However, these radiological findings manifest in varying degrees and may be absent or vague on radiological examination [10,11]. In this case, the patient presented with a two-week history of malaise, chills, and shortness of breath and one-day history of polyarthritis, limiting his ambulation. Radiological examination showed large knee joint effusion; however, no linear calcification of the articular cartilage was seen. Knee arthrocentesis was performed, which revealed monosodium urate (MSU) and calcium pyrophosphate dehydrate (CPPD) crystals using compensated polarized microscopy. Despite the widespread use of polarized light microscopy, proper crystal identification poses technical hurdles, and microscopist expertise is essential. A survey of 110 rheumatologists, laboratory workers, and physicians was conducted online. Participants were shown slide pictures of several crystals to identify. Only one in three participants was able to identify all the CPPD crystals and one in 20 participants could not identify any typical picture of CPPD at all [12]. The technical difficulties in identifying pseudogout under polarized light microscopy are highlighted in this work since both its shape and faint positive birefringence might be difficult to discern. Given this data, cases of concurrent gout and pseudogout may be under-reported due to diagnostic difficulties. This case demonstrates an unusual situation where the patient's arthritis was secondary to three primary mechanisms.

## Conclusions

This case illustrates the first report of gout, pseudogout, and ReA in the same joint. While ReA is typically associated with genitourinary or gastrointestinal infections, it is also important to consider it in the context of pulmonary infection with *C. pneumoniae*. This case emphasizes the importance of combining a clinical history with pathological evaluation before establishing a final diagnosis.
